# From FISH to Hi-C: The Chromatin Architecture of the Chromosomal Region 7q36.3, Frequently Rearranged in Leukemic Cells, Is Evolutionary Conserved

**DOI:** 10.3390/ijms22052338

**Published:** 2021-02-26

**Authors:** Gesualda M. Gulino, Francesca Bruno, Valentina Sturiale, Desiree Brancato, Denise Ragusa, Sabrina Tosi, Salvatore Saccone, Concetta Federico

**Affiliations:** 1 Department Biological, Geological and Environmental Sciences, University of Catania. Via Androne 81, 95124 Catania, Italy; gesualda.gulino@gmail.com (G.M.G.); francesca.bruno@unict.it (F.B.); valentina.sturiale@unict.it (V.S.); desiree.brancato@phd.unict.it (D.B.); 2 Division of Biosciences, College of Health and Life Sciences, Brunel University London, Uxbridge UB8 3PH, UK; denise.ragusa2@brunel.ac.uk (D.R.); sabrina.tosi@brunel.ac.uk (S.T.)

**Keywords:** *MNX1* gene, chromatin loops, neuronal differentiation, topologically associated domains, human lymphocytes

## Abstract

Fluorescence in situ hybridization (FISH) and Hi-C methods are largely used to investigate the three-dimensional organization of the genome in the cell nucleus and are applied here to study the organization of genes (*LMBR1*, *NOM1*, *MNX1*, *UBE3C*, *PTPRN2*) localized in the human 7q36.3 band. This region contains the *MNX1* gene, which is normally not expressed in human lymphocytes beyond embryonic development. However, this homeobox gene is frequently activated in leukemic cells and its expression is associated with an altered gene positioning in the leukemia cell nuclei. In this study, we used FISH on 3D-preserved nuclei to investigate the nuclear positioning of *MNX1* in the leukemia-derived cell line K562. Of the five copies of the *MNX1* gene present in K562, four alleles were positioned in the nuclear periphery and only one in the nuclear interior. Using the Juicebox’s Hi-C dataset, we identified five chromatin loops in the 7q36.3 band, with different extensions related to the size and orientation of the genes located here, and independent from their expression levels. We identified similar loops in 11 human and three mouse cell lines, showing that these loops are highly conserved in different human cell lines and during evolution. Moreover, the chromatin loop organization is well conserved also during neuronal cell differentiation, showing consistency in genomic organization of this region in development. In this report, we show that FISH and Hi-C are two different approaches that complement one another and together give complete information on the nuclear organization of specific chromosomal regions in different conditions, including cellular differentiation and genetic diseases.

## 1. Introduction

Several techniques have been developed to investigate the genome organization at different levels, including molecular cytogenetic methods, such as fluorescence in situ hybridization (FISH), and other molecular approaches, such as chromosome conformation capture (3C) and its derivatives chromosome conformation capture-on chip (4C) and chromosome conformation capture carbon copy (5C), or high-throughput chromosome conformation capture (Hi-C). These latter molecular techniques share a common basic procedural step, even if they show differences in the spectrum of analyzable results. Among these, Hi-C is a high-throughput method that probes the three-dimensional architecture of whole genomes by coupling proximity-based ligation with massively parallel sequencing [1,2].

The topological organization of the human genome in the nucleus plays a key role in several functional properties, such as the transcriptional control of genes [3,4], and Hi-C analyses showed how regulatory elements, such as enhancers, can control many genes located even hundreds of kb away. Although it remains to be clarified how the selective interaction of enhancers occurs with their respective target genes, it is clear that the genome organization in interaction domains separated by specific border regions is a very important feature in the genome functionality. Indeed, Hi-C study showed that the genome is partitioned in specific structures called “topologically associated domains” (TADs), hundreds of kilobases to several million bases long, that are stable for many cell divisions, invariant across different cell types, and evolutionarily conserved in related species [5]. Because of the high degree of conservation, TADs have been considered as the basic units of chromosome folding and an essential structure in chromosome organization [6]. The Hi-C data also showed that TADs are partitioned into long-range contact patterns belonging to two compartments, called A and B, with the former highly enriched of open chromatin, and the second of closed chromatin [1]. Later, it was highlighted a partitioning in at least five sub-compartments, with two of these, namely A1 and A2, correlated with the previously described compartment A. Both A1 and A2 are gene-dense, have highly expressed genes, harbor activating chromatin markers such as H3K36me3 and H3K79me2, and are not close to the nuclear lamina. The other three sub-compartments, labeled B1, B2, and B3, are correlated with compartment B, showing opposite properties with respect to A1/A2 [7]. The compartment A and B correspond to the previously identified (by in situ hybridization of the H3 and L1 isochore families) gene-dense and gene-poor chromosomal regions, respectively, occupying the more internal and the more peripheral nuclear compartment, respectively [4,8]. Other studies indicate a planar supramolecular structure of chromatin characterized by the dynamic properties and multiple functions of DNA in chromosomes and in interphase nuclei. The planar chromatin model proposes that chromatin is arranged in multiple layers capable of stacking in two states; the active state is characterized by an unstacked layer, while the inactive by a stacked layer [9], with these two alternative state layers presumably corresponding to the above compartments A and B, respectively.

TADs represent the basic unit of chromosomal folding; therefore, they play important roles in transcriptional regulation, DNA replication, and other processes involving chromatin organization [10]. They are not only physically isolated units of the genome, but they also represent functionally isolated regulatory domains where enhancers and promoters can interact. Indeed, genes in TADs can be co-regulated, meaning that TADs organize themselves into autonomous regulating domains, and that genes located in the same TAD share similar expression profiles among different cell types [11]. However, TADs are quite different from bacterial operons, in which all genes within are activated concurrently, as co-regulation can occur only on a subset of genes within each TAD [12]. Most likely, the ability to restrict co-regulation only to genes within a TAD is maintained by the activity of the border regions of each TAD. In fact, it has been shown that deletion of the boundary regions leads to ectopic activation of genes both in cell cultures and in vivo. As a result, today, it is thought that many diseases, including cancer, are due to the destruction of the TAD structure. For example, deletion, inversion, or duplication of TAD borders are considered the cause of the ectopic expression of many regulatory genes of limb development, and are the cause of familiar forms of polydactyly [13].

Many studies report alterations in the genome of cancer cells occurring as the result of an “enhancer hijacking” towards a gene different from its target, due to the elimination of the border regions of TADs that normally behave like a barrier. For example, this occurs in acute myeloid leukemia (AML) associated with a recurrent inversion of chromosome 3 [14]. A useful tool for the study of leukemia biology is the cell line K562, derived from chronic myeloid leukemia (CML) patients in blast crisis [15]. K562 cells have four copies of chromosome 7, three with normal morphology and one with a duplication of the 7q36 region repositioned at the end of its short arm [16]. The duplicated region contains the *MNX1* (Motor Neuron and Pancreas Homeobox 1) gene, also known as *HLXB9*, a homeotic gene involved in the development of the body’s structural plan during embryonic development. It has three exons and encodes the transcription factor HB9, which presents the homeodomain typical of DNA-binding proteins [17]. As previously described, *MNX1* is normally expressed only during embryonic development but not in adulthood. In contrast, in a rare subtype of pediatric leukemia, this gene shows an ectopic expression in the leukemic cells, and this seems to be associated with its repositioning inside the nuclear space. The *MNX1* gene is normally localized towards the nuclear periphery in whole blood lymphocytes, in association with lack of expression. However, in leukemia patients, *MNX1* is re-positioned towards the inner part of the leukemic cell nuclei, a compartment where genes are generally expressed [18].

Here we show the structural and functional organization of the genes *LMBR1*, *NOM1*, *MNX1*, *UBE3C*, and *PTPRN2*, localized in the telomeric band 7q36.3. We used FISH on cells with preserved 3D large-scale chromatin structure to study the topographic organization of these loci in the nucleus of the K562 cells, and the high resolution Hi-C method, in human and mouse cell lines, to obtain information on TAD organization of the region. Results highlighted a nuclear repositioning of one of the five *MNX1* alleles present in the K562 cells, coherent with its high expression level in this cell line, and five chromatin loops in both human and mouse cells, thus also demonstrating the evolutionary conserved organization of TADs identified in the 7q36.3 chromosomal band.

## 2. Results

### 2.1. Genomic Organization and Transcriptional Properties of the Gene Cluster in Human 7q36.3 Band

**Figure 1:**
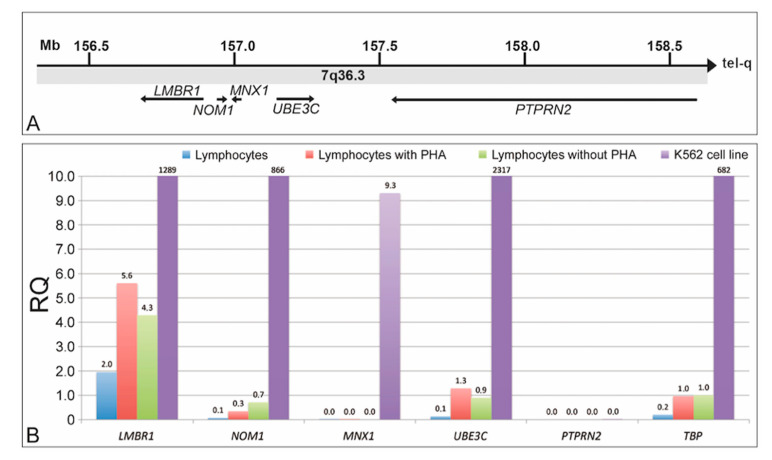
Genomic organization, and expression level of the gene cluster located in the human 7q36.3 chromosomal band. (**A**) Size, position, and transcriptional direction of *LMBR1*, *NOM1*, *MNX1*, *UBE3C*, and *PTPRN2* genes. (**B**) Expression level of the above genes in human whole blood lymphocytes uncultured, cultured with and without PHA, and in the K562 cell line.

The *MNX1* gene is located at the telomeric end of the long arm of the human chromosome 7, close to *LMBR1*, *NOM1*, *UBE3C,* and *PTPRN2* genes, with the first two located towards the centromeric side and the other two towards the telomeric end of the chromosome, in respect to *MNX1* (Figure 1A). Among these genes, *MNX1* is the smallest one, and *PTPRN2* is the largest, showing a genomic size of over 1 Mb.

The expression level of these genes, obtained by qRT-PCR with specific primers (Table 1), showed that in whole blood lymphocytes, the expression level is low or absent for all the analyzed genes. In lymphocytes cultured with or without PHA, *LMBR1*, *NOM1,* and *UBE3C* increased their expression level, while *MNX1* and *PTPRN2* remained inactive. In leukemic-derived K562 cell line, all the analyzed genes, except *PTPRN2*, showed a high level of expression (Figure 1B).

Concerning *MNX1* gene, the results agree with previous studies showing that this gene is transcriptionally inactive in lymphocytes of healthy donors, but highly expressed in subjects with myeloid leukemia [18] and in cells derived from leukemic subjects, as shown here in the cell line K562 where the gene is transcriptionally active. Several studies have shown that *MNX1* is active only during embryonic development, and that in adults with leukemia, it is ectopically expressed in association with a re-localization towards the inner part of the nucleus, triggering the expression of one of the two alleles as a consequence of a chromosomal rearrangement involving the chromosome 7 [18,19].

### 2.2. The 7q36.3 Chromosomal Band in the Human K562 Cell Line

**Figure 2:**
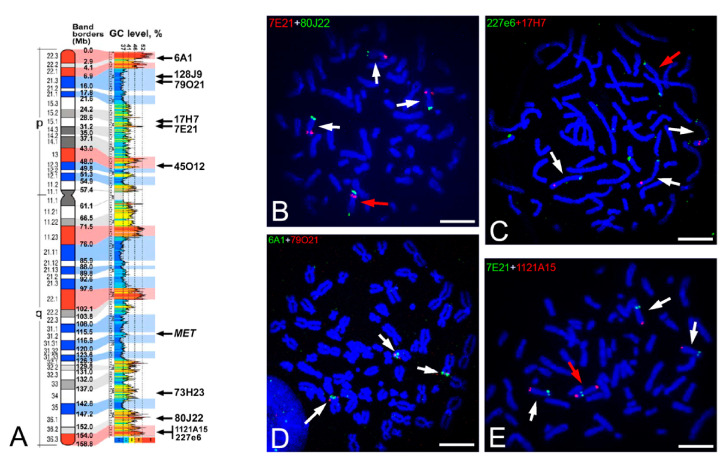
Characterization of the rearranged chromosome 7 in the K562 cell line. (**A**) Ideogram of the human chromosome 7 showing from left to right: the chromosomal bands, the position of each band border with respect to the telomeric end of the p-arm, the GC-level profile along the chromosome, and the position of the BAC probes used in the present work (see probe details in the material and methods section). Red and blue staining indicate the GC-richest and the GC-poorest regions, respectively [8,21]. (**B**–**E**) Representative metaphase plates from K562 cells showing the location by in situ hybridization of some probes, as examples, used in the present work. Scale bars 5 µm. The red or green staining of the probe name indicated at the upper left of each image is related to the fluorochrome used for detection: fluorescein (green signals) or rhodamine (red signals). Chromosomes are stained with DAPI (blue). The red and white arrows indicate the rearranged and non-rearranged chromosome 7, respectively. In the image (**D**), the rearranged chromosome 7 is not visualized, because the two used probes RP11-6A1 and RP11-79O21 are located in the deleted segment.

K562 is a leukemic-derived cell line with four chromosome 7s, one of them endowed by a deletion/duplication rearrangement involving the 7q36.3 region, where the *MNX1* gene is located [16]. In detail, the distal part of the short arm is deleted, while the distal part of the long arm is duplicated, with the duplicated segment placed at the end of the deleted short arm. We did not detect the rearrangement between chromosome 7 and chromosome 18 described for another K562 clone [20]. To better characterize this rearrangement in the clone of the cell line we used in this work, we carried out in situ hybridizations with a number of probes located along the chromosome 7 (Figure 2). The results showed that loci covered by RP11-6A1, RP11-128J9, RP11-79O21, and RP11-17H7 are not present in the rearranged chromosome 7, and the ones covered by probes RP11-RP11-73H23, RP11-80J22, RP5-1121A15, and 227e6 have been observed in the rearranged short arm (see some examples in Figure 2). This indicates that the breakpoint in the short arm is located between the RP11-17H7 and RP11-7E21 probes. Moreover, the breakpoint in the long arm was positioned between *MET* and RP11-73H23 loci, with this latter result in agreement with previous data [16]. 

Considering the above results, the 7q36.3 region in the K562 cells used in this work is represented five times, four of which are in the normal position at the telomeric end of the long arm of the four chromosomes 7, and an additional time in the rearranged chromosome 7, at the end of the short arm, where conversely a large part of this chromosomal arm is absent, thus determining three copies of this deleted region in the K562 cells. 

### 2.3. Radial Nuclear Location of the MNX1 Alleles in the K562 Cells

**Figure 3:**
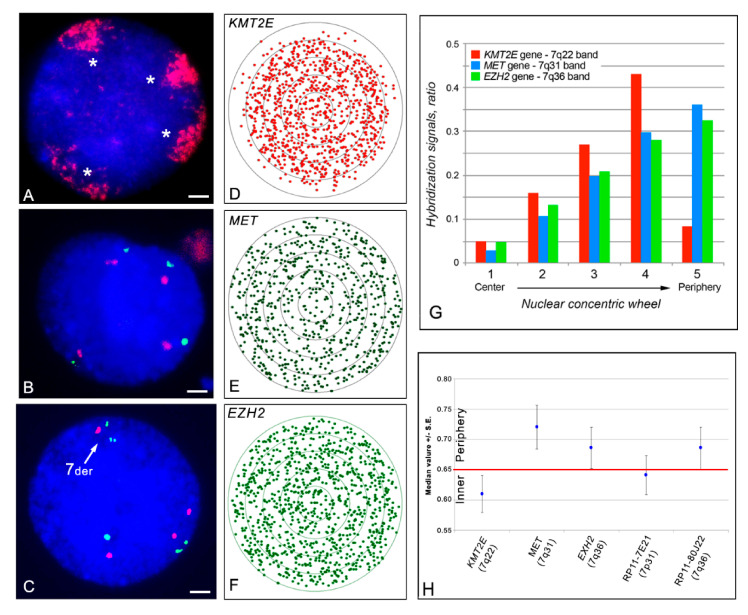
Chromosome 7 in K562 cell nuclei. (**A**) Visualization of the four chromosome territories present in the K562 cells by in situ hybridization with a chromosome painting probe detected by red signals. The stars indicate the four chromosome 7 territories. (**B**) Nuclear location, by in situ hybridization, of the probes *KMT2E* and *MET* located in the chromosomal bands 7q22 (red signals), and 7q31 (green signals), respectively. (**C**) Nuclear location, by in situ hybridization, of the probes *KMT2E* and *EZH2* located in the chromosomal bands 7q22 (red signals), and 7q36 (green signals), respectively. 7der and the white arrow indicate the rearranged chromosome 7 present in the K562 cell line. Nuclei were stained with DAPI (blue). Scale bars are 2 µm (**D**–**F**) Distribution of all the signals detected with the indicated probes and statistically analyzed as previously described [8]. (**G**) Histogram showing the signal distribution presented in (**D**–**F**), respectively, considering five concentric circles denoting nuclear sections. Note the very low number of signals in the more peripheral sector with the *KMT2E* gene probe. (**H**) Radial nuclear location of the above three probes (left part of the graph) evaluated by the median value and the relative standard error (S.E.). The median value of all the detected signals was 0.610, 0.721, and 0.687 for the *KMT2E*, *MET*, and *EZH2* gene probes, respectively. On the right part of the graph, the radial nuclear location of two BAC probes RP11-7E21 and RP11-80J22 are shown, with the former close to the centromeric part of the breakpoint in the short arm of the rearranged chromosome 7.

Hybridization with a painting probe on K562 cell nuclei showed a general nuclear peripheral location of the four chromosome 7 territories (Figure 3A), but the different regions were distributed in the nuclei according to the different gene density/GC-level. This has been previously described for this chromosome in the nuclei of PHA-stimulated lymphocytes [8], where chromosome 7 largely occupies the peripheral regions of the nucleus, but it is organized in a zig-zag way with the gene-rich and the gene-poor regions located toward the inner part of the nucleus or at the nuclear periphery, respectively. Indeed, the *KMT2E* probe (7q22 band) showed a radial distribution of the hybridization signals with a median value of 0.616, indicating a location in the inner part of the nucleus. The *MET* probe (7q31 band) showed a radial distribution of the hybridization signals with a median value of 0.724, indicating a very peripheral location, while the *EZH2* probe (7q36 band) showed a median value of 0.687, indicating an intermediate position in the nucleus (Figure 3). In Figure 3B,C, the position of the *KMT2E* gene is clearly visible in a more internal position in the nucleus in respect to the *MET* and *EZH2* genes.

The in situ hybridizations in the K562 cell nuclei with specific combinations of BAC probes within the 7q36 region allowed us to distinguish the rearranged chromosome 7 from the three not rearranged ones (see the example shown in Figure 3C). This analysis suggested that the 7q36.3 region detected by the probes RP11-80J22, RP5-1121A15, and 227e6 (present in the rearranged short arm of one chromosome 7), is located more internally in the nucleus when compared to the canonical allele located in the long arm. Nonetheless, this trend was observed in a limited number of nuclei, and was deemed not statistically significant. Interestingly, however, we also observed asynchrony in the replication timing of these alleles by detecting singlet (one signal) or doublet (two signals) status of the hybridization signals in the nuclei (data not shown), supporting the correlation between the localization in the inner part of the nucleus and transcriptional activity. In fact, the asynchrony in the replication timing of two alleles is strictly related to the expression of the allele, with the active allele replicated early and the not expressed one replicated late during the S phase of the cell cycle [22]. Thus, we have results that the rearranged chromosome 7 contains one *MNX1* allele replicated early and the other replicated late, a strong indication that one of these is located in the inner part of the nucleus.

### 2.4. Contact-Map and TAD Organization of the Human 7q36.3 Region

**Figure 4:**
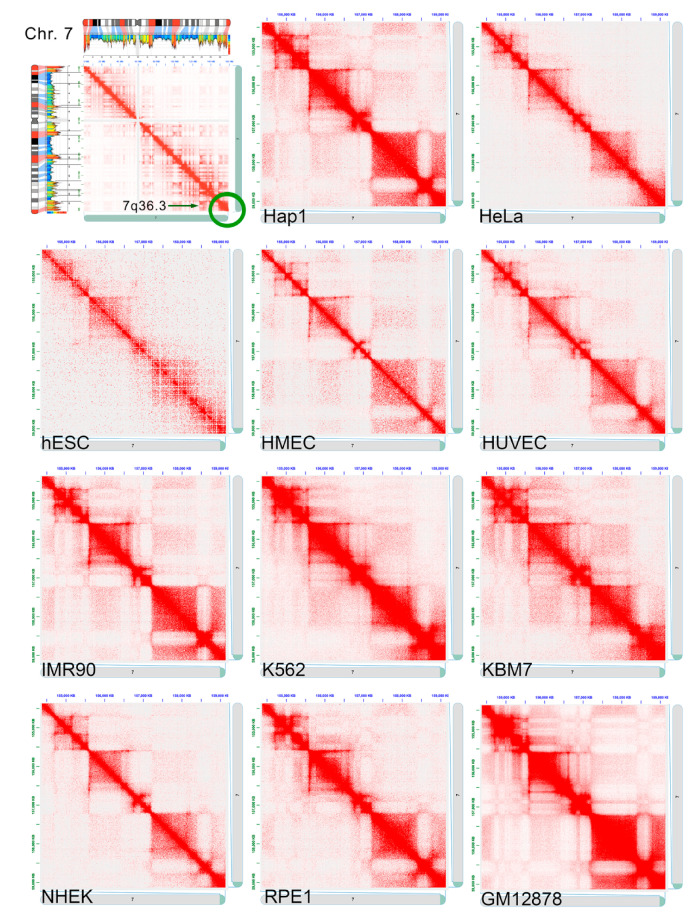
Heat maps of the chromosomal band 7q36.3 in different cell types. Upper left: heat map, at the resolution of 500 kb, of the chromosome 7 in the K562 cell line (data from ref. [7]). Red and blue regions, in the ideogram of the chromosome 7, indicate the GC-rich and the GC-poor bands, respectively (from ref. [8]). The green circle indicates the chromosomal region described in more detail in the other images. The other images show the contact map, at the resolution of 5 kb, of the 7q36.3 band DNA segment observed in a number of different cell types: *Hap1*: near haploid human chronic myelogenous leukemia; *HeLa*: human cervical carcinoma; *hESC*: human embryonic stem cells; *HMEC*: human mammary epithelial; *HUVEC*: human umbilical vein endothelial; *IMR-90*: human lung fibroblasts; *K562*: human erythroleukemia; *KBM7*: near haploid human myelogenous leukemia; *NHEK*: normal human epidermal keratinocytes; *RPE1*: human retinal pigmented epithelial, *GM12878*: human B-lymphoblastoids. Data from ref. [23] (*Hap1*), [5] (*hESC*), [24] (*RPE1*), and 7 (the other cell lines).

We analyzed the chromosomal region 7q36.3 using Hi-C data from different cell lines. Preliminarily, we analyzed the chromatin interactions of the entire human chromosome 7 at the resolution of 500 Kb, and the correlation with the corresponding compositional profile (Figure 4). The GC-rich/gene-rich bands interact not only with themselves, as indicated by the high number of contact close to the diagonal of the heat map, but also with other GC-rich/gene-rich bands with a long-range interaction. This is explainable because the GC-rich bands are localized in the inner part of the nucleus and have an open chromatin organization that allows greater interaction with other GC-rich bands, in agreement with the zig-zag organization of the chromosome 7 previously described [8]. Therefore, the topological structure that can be highlighted by Hi-C further confirms the zig-zag organization of the chromosome 7 territory in the nucleus.

**Figure 5:**
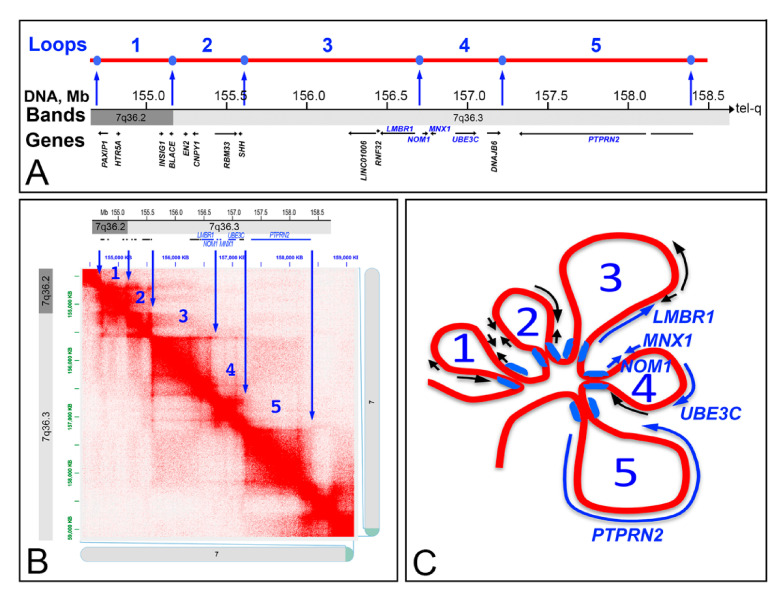
Heat map of the 7q36.3 region showing the number of interactions among DNA segment of 5 kb in size in the K562 cell line. (**A**) Genomic organization of the terminal end of the chromosome 7. The blue spots indicate the position of the CTCF/cohesins, and numbers 1 to 5 represent the five identified chromatin loops. Genes located in the region are also indicated. (**B**) Heat map of the 7q36.3 band in K562 cells (Hi-C data from ref. [7]). The blue arrows indicate the position of CTCF/cohesins, corresponding to the transition point between two adjacent loops. The number of loops (blue numbers) are correlated to the regions indicated in (**A**). (**C**) Loops identified in the 7q36.3 region. The size of each loop reflects the corresponding genomic size, and the genes with their transcriptional orientation are indicated in each loop. The blue symbols at the basis of the loops indicate the position of the CTCF/cohesins.

The heat maps of the chromosomal region 7q36.3, at a resolution of 5 kb, in eleven different human cell lines, showed a high level of similarity, as indicated by the presence of a number of chromatin loops ranging from 3 to 5 (Figure 4). A more detailed description of the chromatin organization of this region was reconstructed according to the heat-map obtained from the K562 cells (Figure 5). In detail, five chromatin loops could be identified, with the third and the fifth being the larger, and the fourth the smallest. The five genes here considered are located in the third loop (*LMBR1* gene), the fourth loop (*NOM1*, *MNX1*, and *UBE3C* genes), and the fifth loop (*PTPRN2* gene) with this latter containing the entire large gene *PTPRN2*.

### 2.5. Contact-Map of the Mouse Regions Syntenic to the Human 7q36.3 Region

To verify if different species maintained the same loop organization of the chromosomal region 7q36.3, we analyzed the heat map of the syntenic region in the mouse chromosomes containing the *MNX1* gene (Figure 5). In particular, we highlighted that during cell differentiation, the TAD structure is not altered. However, in the heat map of embryonic stem (ES) cells, there are two regions with a higher intensity of red color (flagged by blue arrows), which indicates a greater number of interactions. These regions represent the interaction between *Mnx1* and two other genes (*En2* and *Shh*) also involved in embryonic development. The disappearance of these regions during cell differentiation suggests a possible coordination of the activity of the *Mnx1*, *Shh*, and *En2* genes in ES cells, likely mediated by the structural organization of the loop.

**Figure 6:**
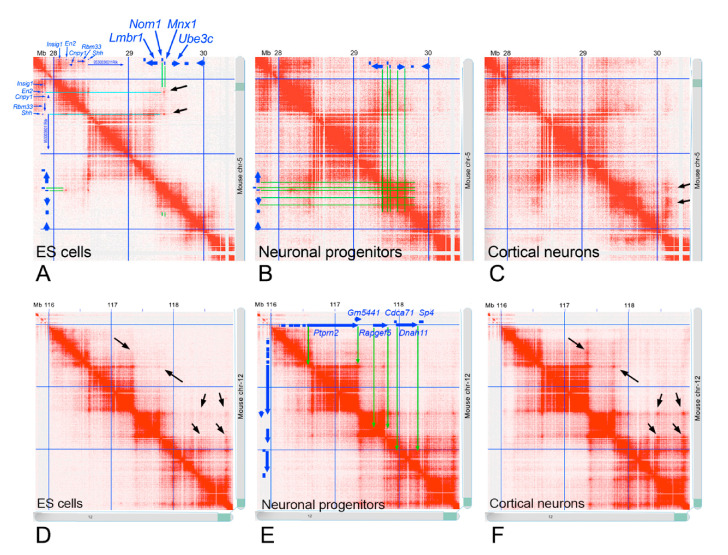
Heat map of the mouse syntenic region to the 7q36.3 human chromosomal band. (**A**–**C**) Heat map of the mouse chromosome 5 region containing the *Lmbr1*, *Nom1*, *Mnx1*, and *Ube3c* genes, in the ES cells, neuronal progenitor, and cortical neurons, respectively. (**D**–**F**) Heat map of the mouse chromosome 12 region containing the *Ptprn2* genes, in the ES cells, neuronal progenitor, and cortical neurons, respectively. The position of the genes is indicated at the top of the panels (**A**,**B**,**E**), and the green lines and arrows show the location of these genes in the corresponding TAD. Black arrows indicate small regions with differences in the number of contacts during neuronal differentiation. Hi-C data from ref. [26].

**Figure 7:**
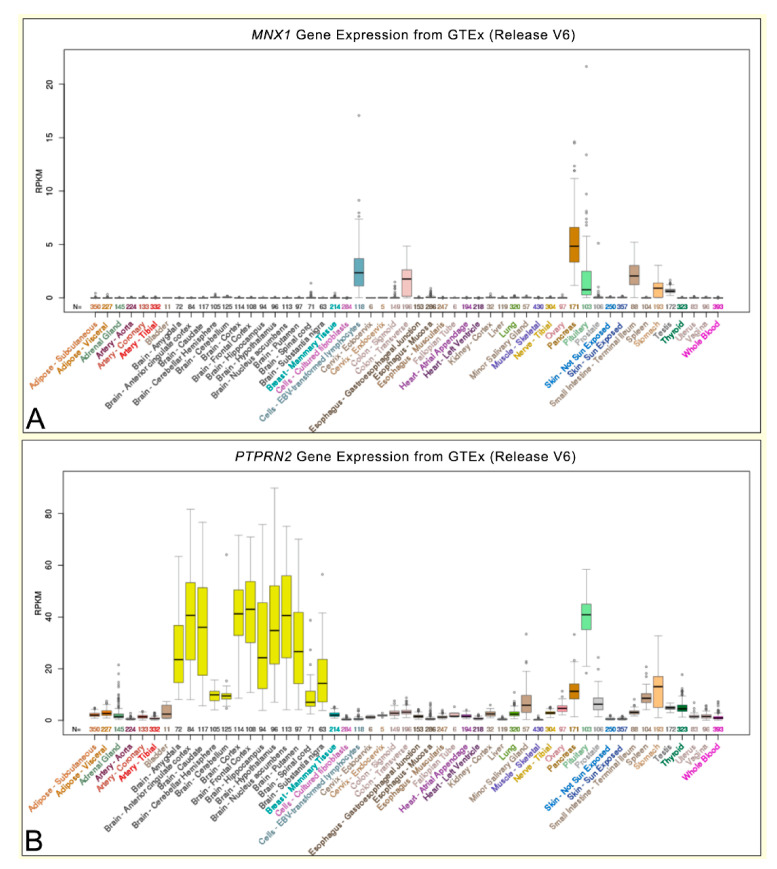
Expression level of the *MNX1* and *PTPRN2* genes in human healthy tissues. (**A**,**B**) Expression of *MNX1* and *PTPRN2* genes, respectively, in 53 human tissues from GTEx RNA-seq of 8555 samples. Data and images from the UCSC Genome Browser (http://genome.ucsc.edu, accessed on 16 June 2018).

In the mouse chromosomes, our analyzed gene cluster is not located in a single region, but is split in two parts, one in chromosome 12, containing the *Ptprn2* gene, and the other in chromosome 5, containing the *Lmbr1*, *Nom1*, *Mnx1*, and *Ube3c* genes. In chromosome 12, the *Ptprn2* gene spans an entire large loop (Figure 6), as observed in the human genome. In terms of expression, *PTPRN2* is highly expressed in human neuronal cells (Figure 7) and its expression levels change during neuronal differentiation [25]. In particular, this gene is not expressed during the embryonic stage, while it exhibits increasingly higher levels of expression during the differentiation of embryonic stem cells first into neuronal progenitors and then into differentiated cortical neurons. The expression of *PTPRN2* does not appear to affect the size of the chromatin loop in the ES cells, in the neuronal progenitor, nor in cortical neurons (Figure 6). However, an increased number of contacts between different loops (indicated by black arrows in Figure 6) is observed through different stages of neuronal cell differentiation. In particular, the promoter region of the *Ptprn2* gene; in fact, some small regions, gradually during the differentiation, acquire an increasingly intense red color as differentiation proceeds, representing a greater number of contacts possibly due to interactions among regulatory elements involved in neuronal cell differentiation.

As for the mouse *Lmbr1*, *Nom1*, *Mnx1*, and *Ube3c* genes, the loop organization presents itself similarly to that observed in the human chromosome 7. Despite the *Lmbr1* gene being located onto a different loop than the other three genes (i.e., *Nom1*, *Mnx1*, and *Ube3c*), they are positioned with the same orientation in respect to the loops and one to each other. The expression of *Mnx1* is high during embryonic development, but decreases to zero in the large number of differentiated cells (Figure 7).

In both the analyzed chromosomal regions, the chromatin loop organization is not affected by cell differentiation. The number and the size remain the same during the developmental progression, with only small regions being detected with a variable number of contacts, possibly due to the differential interactions with regulatory regions.

## 3. Discussion

In this study, we analyzed the *MNX1* gene and its encompassing genomic region at the 7q36.3 chromosomal band. This gene, while not expressed in the whole blood human lymphocytes, is activated in leukemic cells by ectopic repositioning in the cell nucleus of one of its alleles [18,27]. We evaluated the nuclear location of the chromosomal region surrounding *MNX1* in the leukemia K562 cell line by FISH, and assessed the transcriptional activity of genes mapping in same region (i.e., *LMBR1*, *NOM1*, *MNX1*, *UBE3C*, and *PTPRN2)* by qRT-PCR. The K562 cell line used in this work contains four chromosome 7, three of which are normal, and the fourth endowed by a duplication of the terminal part of the long arm, with this duplicated segment positioned in the p arm of the same chromosome, as described in a previous analysis [16]. The four chromosome 7 territories of K562 cells are observed at the nuclear periphery, namely in the same compartment previously described in the human normal lymphocytes [8], even if in the rearranged chromosome 7 the position of one *MNX1* allele was detected more internally in the nucleus. On the other hand, with Hi-C analysis in the K562, we did not detect appreciable differences in the contact map of the region where *MNX1* is localized, with respect to the other cell lines (Figure 4). While the repositioning of an *MNX1* allele in the nucleus is coherent with the overexpression of *MNX1* in the K562 cell line, no change in the TAD organization was detectable by the Hi-C analysis. Even if the allele reposition in interphase nucleus is associated with its transcriptional activation, Hi-C does not seem to be able to clearly highlight these changes, at the resolution level used; thus, an additional FISH analysis with specific gene probes can be useful to evaluate the whole chromatin organization.

*LMBR1*, *NOM1*, *MNX1*, *UBE3C*, and *PTPRN2* are all located in defined DNA loops that can contain both active and inactive genes. In fact, the expression status of these genes does not seem to be related to the loop features. We demonstrated this in K562 cells, where the expression of *MNX1* and *PTPRN2* genes was high or undetectable, respectively, and during neuronal differentiation of mouse cells, where the same genes were expressed in opposite ways, without detectable alterations of the DNA loop organization. The changes observed during cell differentiation are usually associated to additional interactions with specific regions also present in different loops, possibly due to the presence of very distant control regions, even if they are in different chromosomes [28,29], or to a transcriptional co-regulation of some genes, as observed for example for *Mnx1* and *Shh* genes in the mouse stem cells, in accordance with previous reports [30]. Indeed, it was described that a great part of non-coding regions is biochemically active, contributing to the correct regulation of gene expression, and that these regulatory domains could determine human diseases when their structure is disrupted [28]. Moreover, regulatory domains could be located on different chromosome territories, with respect to the location of the regulated gene. This indicates a direct association between structural chromosomal aberrations and alterations in nuclear architecture, which represents as a possible molecular pathogenetic mechanism in the development of diseases. For instance, chromosomal interactions and chromatin architecture were shown to be a relevant feature of the genetic disorder 2q37-deletion syndrome, as demonstrated by studies with DNA-FISH and Hi-C on the differences in interactions between chromosomes 2q, 12 and 17 in human mesenchymal stem cells (MSCs), and MSC-derived cell types of families with 2q37-deletion [29]. In our case, we did not detect loss/gain of chromatin interactions of the 7q36.3 region with other chromosomal regions, in the K562 cells. Therefore, the overexpression of *MNX1* gene in this cell line could be due to other, yet not be identified, factors, but certainly related to the altered nuclear position detected by FISH.

Thus, considering the loop containing the *MNX1* gene, it is of interest to note the absence of reorganization following the repositioning of one of the *MNX1* alleles to the inner part of the nuclei, not detectable by Hi-C, but associated with an increased level of expression. Therefore, we may deduce that loop size and gene location into a loop are two features of chromatin organization that are strictly regulated but independent from gene expression. This further confirms that gene expression is finely regulated by a number of genomic properties, which are not only limited to the structural organization of the TAD where the genes are located, as it was previously reported for the *Shh* gene in mouse, showing a correct pattern of expression even if TAD boundaries are disrupted [30].

We also confirm that this organization is evolutionary conserved, as the same type of chromatin loops organization of the human 7q36.3 region is observed in the syntenic region of the mouse chromosomes, and the region resulted split into two different chromosomes. However, this is not in contrast with the presence of co-regulated elements located in different loops of this region, interactions being always possible between these sequences, even if they are present in different chromosomes.

## 4. Materials and Methods

### 4.1. Cell Cultures

Human leukemia K562 cell line (from Biological Bank and Cell Factory IST, Genova, Italy, code No. HTL94001) was grown at 37 °C and 5% CO_2_, in RPMI 1640 supplemented with 15% FBS, 1% penicillin/streptomycin, and 1% L-glutamine [16]. Human lymphocytes were obtained from whole peripheral blood samples of healthy volunteers, and cell separation with Ficoll solution. Phytohaemagglutinin (PHA) stimulated lymphocytes were then obtained by 72-h cultures in RPMI 1640 supplemented with 20% FBS, 1% penicillin/streptomycin, 1% L-glutamine, 3% PHA at 37 °C, and 5% CO_2_. All procedures involving human participants were in accordance with the ethical standards of the institutional and/or national research committees and with the 1964 Helsinki declaration and its later amendments or comparable ethical standards. Informed consent was obtained from the volunteer donors. Ethical approval no: 16516-TISS-Apr/2019-18741-2.

### 4.2. In Situ Hybridization

To prepare metaphase chromosomes, colcemid (0.05 μg/mL) was added to cell cultures 1 hour before harvesting. Then, cells were harvested in hypotonic solution (sodium citrate 1%) and fixed with methanol-acetic acid (ratio 3:1). Interphase nuclei were also obtained using a protocol to preserve the 3D chromatin structure, as previously described [8]. FISH experiments were performed using probes specific for loci along the chromosomes 7. BACs RP11-90N9 (GenBank accession No. AZ518618.1), RP11-213E22 (GenBank accession No. AQ484445.1), RP11-6A1 (GenBank accession No. AC006433.18), RP11-128J9 (GenBank accession No. CL983275.1), RP11-79O21 (GenBank accession No. AQ317691.1), RP11-17H7 (GenBank accession No. AZ515887.1), RP-117E21 (placed in the chromosome at position 30,180,910: from NCBI35/hg17 assembly), RP11-45O12 (GenBank accession No. AQ194455.1), RP11-73H23 (GenBank accession No. AQ266610.1), RP11-80J22 (GenBank accession No. AQ317788.1), and PAC RP5-1121A15 (GenBank accession No. AC006357.5). We also used commercially available probe mixtures XL 7q22/7q36, containing the *KMT2E* and *EZH2* genes, and XL 7q22/q31, containing the *KMT2E* and *MET* genes (MetaSystems^TM^, Althlussein, Germany), and chromosome 7 paint directly labelled in red (Cambio, Cambridge, UK). The RP5-1121A15 clone contains the entire *MNX1* gene [18]. Probe 227e6 is a cosmid clone previously described [18].

Each human DNA sequence inserted in these BAC/PAC clones was extracted from bacteria using a commercial kit (Qiagen, Milan, Italy), digoxigenin or biotin-labelled by nick translation (Roche, Mannheim, Germany), and hybridized as previously described [27]. Detection was carried out using rhodamine conjugated avidin (for biotin-labelled probes), and anti-digoxigenin secondary antibody conjugated with fluorescein (for digoxigenin-labelled probes).

Hybridization signals on metaphase chromosomes and interphase nuclei were analyzed using an Olympus AX70 fluorescence microscope and captured with a charge coupled device (CCD) camera (COHU 4910 series). Images were recorded using MacProbe v4.3 software (Applied Imaging, Newcastle, UK). Moreover, some images were captured using a CCD Photometrics Sensys camera coupled to and driven by ISIS (Metasystems^TM^, Altlussheim, Germany).

### 4.3. Radial Nuclear Location Analysis

Radial nuclear location (RNL) was determined using the 2D FISH analysis as previously described [8]. Briefly, nuclei were hybridized with labelled BAC probes, and at least 300 hybridized nuclei were randomly acquired, by using an epifluorescence microscopy equipped with a CCD camera, and recorded. Each hybridization signal was localized in the nucleus respect to the center/periphery position with a value corresponding to the ratio of its position relative to the nuclear radius (0 and 1 indicate the center, and the periphery of the nucleus, respectively). This was done using a dedicated software developed at the University of Catania [8]. The assessment of the RNL of each probe was then based on the statistical analysis of at least 300 hybridization signals from the randomly recorded nuclei on at least three different experiments for each probe. RNL was statistically defined as the median value ± confidence interval of the analyzed hybridization signals. It was established, analyzing a high number of probes [8,18,27,31], and other own unpublished data], that median values of 0.65 could be considered a landmark between loci located at the nuclear periphery and at the nuclear interior. Differences in RNLs were statistically evaluated by two-tailed *t*-test. Statistical values and analyses were performed by Microsoft Excel v. 16.44, and StatView v. 1.03 softwares.

### 4.4. Expression Analysis

Total RNA was extracted from K562 cells using the automatic nucleic acid extractor (Mag-Core, Diatech Lab Line, Ancona, Italy). Subsequently, the RNA retrotranscription was performed using the SuperScript™ III First-Strand Synthesis SuperMix (Invitrogen, Thermo Fisher Scientific Italia, Milano, Italy). The obtained cDNA samples were quantified, by quantitative real time PCR (qRT-PCR) experiments, using the SensiFASTTM SYBR® & Fluorescein, Bioline kit (Bioline Reagents Ltd., UK). Primers to detect target and control genes were specifically designed (Table 1). At least three replicates for each gene were performed, and the obtained data were analyzed using the Ct value of each sample. This was normalized with the Ct value of the endogenous control (TBP) obtaining the ΔCt, and after normalization with the calibrator (PHA-stimulated lymphocytes), the obtained ΔΔCt values were finally used to determine the relative quantification (RQ) of each sample, applying the 2^−ΔΔCt^ formula.

### 4.5. Hi-C Data Sets and Analysis

The Hi-C data were obtained from the available archive of studies published between 2009 and 2015 and most of the data refer to those described by Rao et al. [7], in which the resolution of the contact regions reaches levels up to 1 Kb. The evaluation of the number of contacts of the analyzed chromosomal regions was obtained using the visualization software for HiC data, Juicebox version 1.5.2, developed in the lab of Dr. Lieberman Aiden [7,32].

The heat maps, which show the contact regions between the analyzed chromosomal regions, were generated both at low resolution (500 kb) and at high resolution (5 kb) and correlated with a number of genomic properties (chromosomal bands and GC level, genes, epigenetic markers). The position of the chromosomal bands, and of the relative GC level, was obtained from Federico et al. [8]. The other epigenetic features were obtained from the UCSC Genome Browser (http://genome.ucsc.edu, accessed on 16 June 2018).

## 5. Conclusions

FISH and Hi-C can be considered two different approaches that complement one another to study the genome organization in the cell nucleus. The complementary use of FISH and Hi-C allows us to understand the position of defined chromosome territories and loci in the nucleus and to define the level of physical interactions among all the loci of a genome. As chromatin organization is tightly linked to the correct expression of genes, these approaches are valuable in understanding genomic interactions beyond linear nucleotide sequences. Indeed, the use of Hi-C as a unique molecular method fails to give complete structural and functional information on the analyzed genomic regions, as described here for the 7q36.3 region in K562 cells, where one of the five alleles of the *MNX1* gene, presumably responsible for the over-expression of that gene, has a different position in the nucleus than the canonical one, not detectable by Hi-C analysis. Thus, *MNX1* overexpression is possibly related to its repositioning in a different compartment of the cell nucleus, and not to a disruption of the TAD where it is located.

In this study, we integrated FISH on nuclei prepared with a protocol preserving 3D chromatin structure and Hi-C data to uncover the genomic organization of the 7q36.3 chromosomal region in K562 cells. By comparing the arrangement of this region in different human and mouse cell lines, this locus appears to be evolutionarily conserved. During neuronal differentiation, while we observe minor changes in genomic contacts in the region, the overall structural organization remains intact. This suggests that the regulation of genomic structure is independent from the transcriptional status of individual genes.

## Figures and Tables

**Table 1:** Primers used in the present study.

Gene	Nucleotide sequence (5′-3′)	
***LMBR1***	Forward	CATGGTTTGTGGAATCTTGC
	Reverse	GATTCCCTTTTTCAGGCCAG
*NOM1*	Forward	GACCAGGATTCGGTTTATGC
	Reverse	GACCAAAGCTCTCTGCAGTT
*MNX1*	Forward	GTTCAAGCTCAACAAGTACC
	Reverse	GGTTCTGGAACCAAATCTTC
*UBE3C*	Forward	AGGTGCGAGGCAACAAGTTT
	Reverse	GAGAGGGCCCCCAAATAAT
*PTPRN2*	Forward	ATGGAGCACGGATTCATACC
	Reverse	GACGATGGACCTCTTGGTAA
*TBP*	Forward	GCCAAGAGTGAAGAACAG
	Reverse	GAAGTCCAAGAACTTAGCTG

## Data Availability

FISH data are available on request from the corresponding authors. Hi-C data are in the references [5,7,23,24,26], and visible in the Juicebox software version 1.5.2 from the Broad Institute and Aiden Lab (https://aidenlab.org/juicebox/ accessed on 16 June 2018) referenced in [7,32].

## Abbreviations

AML	Acute Myeloid Leukemia
BAC	Bacterial artificial chromosome
CML	Chronic Myeloid Leukemia
CTCF	CCCTC-binding factor
DAPI	4′,6-diamidino-2-phenylindole
En2	Engrailed homeobox 2
EXH2	Enhancer of Zeste 2 Polycomb Repressive Complex 2 Subunit
FISH	Fluorescence in situ hybridization
GTEx	Genotype-Tissue Expression (GTEx)
Hi-C	High-throughput chromosome conformation capture
K562	Leukemia derived cell line
KMT2E	Lysine Methyltransferase 2E
LMBR1	Limb Development Membrane Protein 1
MET	MET Proto-Oncogene, Receptor Tyrosine Kinase
MNX1	Motor Neuron And Pancreas Homeobox 1
NOM1	Nucleolar Protein With MIF4G Domain 1
PHA	Phytohaemagglutinin
PTPRN2	Protein Tyrosine Phosphatase Receptor Type N2
Shh	Sonic Hedgehog Signaling Molecule
TAD	Topologically Associated Domain
TBP	Tata-box binding protein
UBE3C	Ubiquitin Protein Ligase E3C
